# *TMPRSS2-ERG *-specific transcriptional modulation is associated with prostate cancer biomarkers and TGF-β signaling

**DOI:** 10.1186/1471-2407-11-507

**Published:** 2011-12-05

**Authors:** Jan C Brase, Marc Johannes, Heiko Mannsperger, Maria Fälth, Jennifer Metzger, Lukasz A Kacprzyk, Tatjana Andrasiuk, Stephan Gade, Michael Meister, Hüseyin Sirma, Guido Sauter, Ronald Simon, Thorsten Schlomm, Tim Beißbarth, Ulrike Korf, Ruprecht Kuner, Holger Sültmann

**Affiliations:** 1 Unit Cancer Genome Research, Division of Molecular Genetics, German Cancer Research Center and National Center for Tumor Diseases, Im Neuenheimer Feld 460, 69120 Heidelberg, Germany; 2 Division of Molecular Genome Analysis, German Cancer Research Center, Heidelberg, Germany; 3 Translational Research Unit, Thoraxklinik, University of Heidelberg, Heidelberg, Germany; 4 Institute of Pathology, University Medical Center Hamburg-Eppendorf, Hamburg, Germany; 5 Martini-Clinic, Prostate Cancer Center, University Medical Center Hamburg-Eppendorf, Hamburg, Germany; 6 Department Medical Statistics, University Medical Center Göttingen, Göttingen, Germany

**Keywords:** Prostate cancer, *TMPRSS2-ERG*, Gene expression profiling

## Abstract

**Background:**

*TMPRSS2-ERG *gene fusions occur in about 50% of all prostate cancer cases and represent promising markers for molecular subtyping. Although *TMPRSS2-ERG *fusion seems to be a critical event in prostate cancer, the precise functional role in cancer development and progression is still unclear.

**Methods:**

We studied large-scale gene expression profiles in 47 prostate tumor tissue samples and in 48 normal prostate tissue samples taken from the non-suspect area of clinical low-risk tumors using Affymetrix GeneChip Exon 1.0 ST microarrays.

**Results:**

Comparison of gene expression levels among *TMPRSS2-ERG *fusion-positive and negative tumors as well as benign samples demonstrated a distinct transcriptional program induced by the gene fusion event. Well-known biomarkers for prostate cancer detection like *CRISP3 *were found to be associated with the gene fusion status. WNT and TGF-β/BMP signaling pathways were significantly associated with genes upregulated in *TMPRSS2-ERG *fusion-positive tumors.

**Conclusions:**

The *TMPRSS2-ERG *gene fusion results in the modulation of transcriptional patterns and cellular pathways with potential consequences for prostate cancer progression. Well-known biomarkers for prostate cancer detection were found to be associated with the gene fusion. Our results suggest that the fusion status should be considered in retrospective and future studies to assess biomarkers for prostate cancer detection, progression and targeted therapy.

## Background

Prostate cancer is the most frequently diagnosed malignancy and still one of the leading causes of cancer related death in men [1]. Since the discovery of a recurrent gene fusion between the androgen responsive gene *TMPRSS2 *(transmembrane protease, serine 2) and *ERG *(v-ets erythroblastosis virus E26 homolog (avian)) on chromosome 21 [2], prostate cancers are molecularly divided into "fusion-positive" and "fusion-negative" cancers. Although the *TMPRSS2-ERG *fusion is a critical early and common event in prostate cancer development and progression [3,4], the clinical implications of the fusion are controversial [5-9] and the functional consequences are unclear.

After the rearrangement, *ERG *expression is driven by the androgen-responsive promoter of *TMPRSS2*, resulting in a significant upregulation of the transcription factor ERG [2,10]. Initial *in vitro *experiments demonstrated that *ERG *overexpression leads to increased invasion via the induction of metalloproteinase and plasminogen activator pathway genes [11]. The molecular effects of the gene fusion were recently found to be associated with an activation of WNT-signaling which induces epithelial-to-mesenchymal transition (EMT) and loss of cell adhesion [12,13]. Additionally, *ERG *overexpression was shown to modulate androgen receptor signaling and to initiate epigenetic silencing resulting in cellular dedifferentiation [14].

To study the functional consequences of *TMPRSS2-ERG *fusion on the transcriptome level, we analyzed large-scale gene expression profiles using Affymetrix GeneChip Exon 1.0 ST microarrays. Our results demonstrate that the *TMPRSS2-ERG *gene fusion leads to transcriptional modulation, which is associated with widely accepted prostate cancer biomarkers and signaling pathways.

## Methods

### Biological samples

Prostate tissue samples were obtained from the University Medical Center Hamburg Eppendorf. Approval for the study was obtained from the local ethics committee and all patients agreed to additional tissue sampling for scientific purposes. Tissue samples from 47 prostate cancer patients with clinical high-risk tumors were included (Additional file 1: Table S1). None of the patients had been treated with neo-adjuvant radio-, cytotoxic- or endocrine therapy. During radical prostatectomy, tissue samples from the peripheral zone of the prostate were taken with a 6 mm punch biopsy instrument immediately after surgical removal of the prostate from tumorous areas as described before [15]. The punches were immersed in RNAlater (Qiagen, Hilden, Germany) for 24 h at room temperature and subsequently stored at -80°C. To confirm the presence of tumor, all punches were sectioned, and the tumor cell content was determined in every 10th section. Only sections containing at least 70% tumor cells were included in the study. Normal prostate tissue samples from non-suspect areas of the peripheral zone were obtained similarly from 48 different patients with clinical low-risk tumors who underwent radical prostatectomy. These punches were also sectioned and inspected for the presence of normal prostatic epithelial cells in every 10th section. Only sections containing between 20% and 40% normal prostatic epithelial cells were included in the study.

### RNA extraction

Total RNA was extracted using the AllPrep DNA/RNA Mini kit (Qiagen) according to the manufacturer's instructions. Briefly, tissue sections were homogenized in 1 ml RLT Plus buffer using TissueLyser (Qiagen). After DNA separation, 1.5 vol. of 100% ethanol were added to the total RNA and the mixture was purified. The quantity and quality of the total RNA was checked using the Nanodrop photometer (Peqlab, Erlangen, Germany) and the Bioanalyzer (Agilent, Böblingen, Germany). Samples with low RNA quality (RIN < 6) were excluded from further analysis.

### Expression profiling using affymetrix GeneChip exon 1.0 ST arrays

The Affymetrix (Santa Clara, USA) GeneChip Whole Transcript Sense Target Labeling Assay was used to generate amplified and labeled sense DNA. Briefly, 1 μg of total RNA was used for rRNA reduction. Following the manufacturer's instructions, cDNA was hybridized to the Affymetrix 1.0 Human Exon ST arrays and incubated at 45°C for 16 h. The washing and staining steps were carried out using the GeneChip Fluidics station FS 450. Slides were scanned with the Affymetrix Gene Chip scanner 3,000 7 G system.

### Validation of *TMPRSS2-ERG *fusion events

*TMPRSS2-ERG *fusion events were verified using RT-PCR. cRNA from the Affymetrix Whole Transcript Sense Target Labeling Assay was reversely transcribed. 10 ng of cDNA were used for RT-PCR based validation. Initial amplification as well as nested PCR was done using primers described by Jhavar et al. [16]. All products were separated by agarose gel electrophoresis. Additionally, the *TMPRSS2-ERG *fusion transcript was quantified in clinical prostate samples by Taqman (Life Technologies, Carlsbad, USA) real-time PCR using the equivalent of 10 ng as a template. Quantification was performed with 2 × ABSOLUTE QPCR Mix (Thermo Scientific) on the LightCycler 480 System (Roche) using the assay described by Mertz et al. [10]. The amounts of *ERG *(Hs01554635_m1)*, CRISP3 *(Hs00195988_m1) and *TDRD1 *(Hs00229805_m1) transcripts were determined relative to *B2M *(Hs99999907_m1) using the second derivative maximum method of the LightCycler software (validation results are summarized in Additional file 2: Table S2).

### Statistical analysis

Gene expression data from the Affymetrix cel files were analyzed using the statistical computing environment R http://www.cran.r-project.org. Gene expression profiles were obtained by applying the Robust Multichip Average (RMA) [17] implementation included in the Affymetrix Power Tools (APT). The MIAME-compliant microarray data were submitted to the NCBI GEO database (GSE29079). Differentially expressed genes were determined using a moderated t-statistic [18]. All p values were corrected for multiple testing, and genes showing a false discovery rate (FDR [19]) ≤ 0.05 were considered as significantly deregulated. TranscriptCluster IDs of deregulated genes were subjected to pathway exploration using the Ingenuity Pathway Analysis software (Ingenuity, Redwood City, CA). To assess the significant differences of single gene levels, a two-sided Wilcoxon test was used. A *p *value < 0.05 was considered as significant.

For validation purposes, 131 prostate cancer samples from publicly available gene expression data (Taylor et al. [20]) were re-analyzed using the methods described above. Fusion-positive and negative samples were assigned according to their ERG expression levels. Samples with a considerable high ERG expression (48 samples; > 9.2) were labeled as fusion-positive, whereas tumor samples with low ERG expression (72 samples; < 8.0) were marked as fusion-negative. 11 samples with median ERG expression (8.0 < ERG < 9.2) were omitted from the analyses.

## Results and discussion

### *TMPRSS2-ERG *induced transcriptional deregulation

Gene expression profiles were successfully obtained from 47 cancer and 48 normal prostate tissues taken from non-suspect areas of the peripheral zone from clinical low-risk tumors. A limitation for the comparison between tumor and normal was the variability of stromal components and clinical characteristics (Gleason scores/stages) between tumor and normal tissues (see methods).

In a first explorative analysis, we compared the gene expression levels in tumor and normal prostate tissues. Considerable transcriptional deregulation was identified in the malignant tissues: 263 genes had at least a twofold change in expression levels (Additional file 3: Table S3). The list of deregulated genes contained markers that had been described in prostate cancer before (e. g. *CRISP3*, *AMACR *and *MYC*). Additionally, several genes with unknown function like *TDRD1 *and *C20orf74 *were identified as biomarker candidates for prostate cancer.

**Figure 1:**
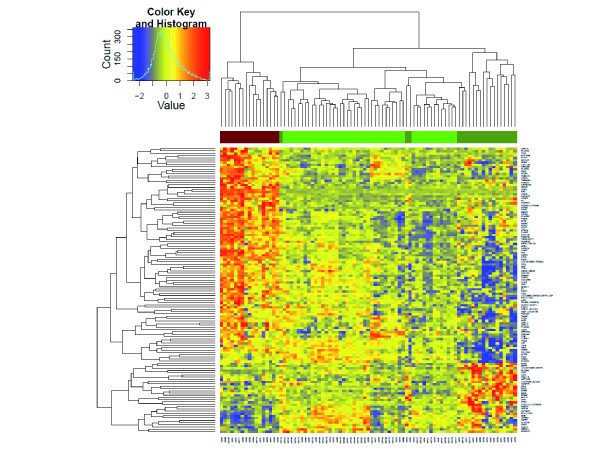
***TMPRSS2-ERG *specific transcriptional modulations**. Clustering of 48 benign (*light green*), 20 fusion-negative (*dark green*) and 17 fusion-positive (*red*) prostate cancer tissue samples. Clustering is based on the expression levels of 126 genes, which showed at least 2-fold expression changes (Additional file 4: Table S4**) **between the *TMPRSS-ERG *positive and negative subgroups. Expression values are color-coded (red = upregulation; blue = downregulation).

To compare the gene expression levels between *TMPRSS2-ERG *fusion-positive and negative tumors, we included only samples (*n *= 37) with reliable assessment based on ERG exons/gene expression levels, nested RT-PCR as well as quantitative RT-PCR measurements (Additional file 2: Table S2). We excluded a group of samples with median *ERG *expression levels, since our initial explorative analysis demonstrated that these samples might lead to noise in subsequent statistical analyses. Seventeen tumor samples were defined as *TMPRSS2-ERG *fusion-positive and 20 samples were defined as *TMPRSS2-ERG *fusion-negative. A total of 1,635 genes was differentially expressed between *TMPRSS2-ERG *positive and negative tumors (FDR < 0.05). The exclusion of the cases with a median ERG expression level might explain the relatively large number of significantly deregulated genes in comparison to other published studies. Roughly 75% of the differentially expressed genes were upregulated in patients with *TMPRSS2-ERG *gene fusion, which is in line with other reports [13,21,22]. A subset of 126 genes showed at least 2-fold expression changes (Additional file 4: Table S4). Clustering of the deregulated genes demonstrated that fusion-negative tumor specimens were more closely related to normal tissue, whereas fusion-positive samples showed distinct transcriptional modulation (Figure 1).

**Figure 2:**
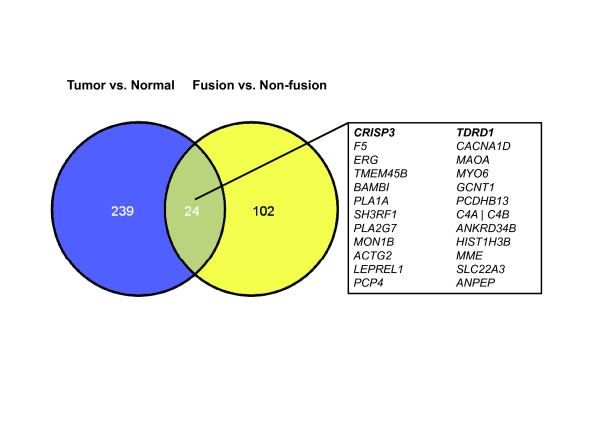
**Overlap of prostate cancer and *TMPRSS2-ERG *biomarkers**. Significant genes with at least 2-fold expression changes in the tumor-normal comparison (blue, Additional file 3: Table S3) and the *TMPRSS2-ERG *subgroups (yellow, Additional file 4: Table S4).

**Figure 3:**
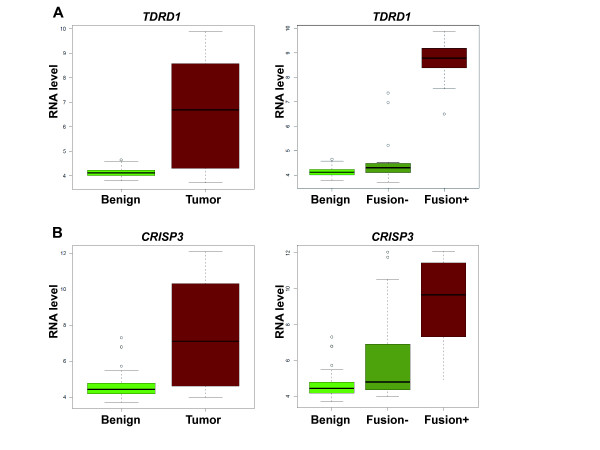
**Association between biomarkers for prostate cancer detection and *TMPRSS2-ERG *gene fusion**. Gene expression of *TDRD1 *(**A**) and *CRISP3 *(**B**) in 48 benign, 47 tumor (*left side*) as well as in the *TMPRSS2-ERG *(20 fusion-negative vs. 17 fusion-positive tumor samples; *right side*).

Of the 126 genes from the *TMPRSS2-ERG *comparison, 24 genes were also found in our initial comparison of expression profiles between tumor and benign tissue samples (Figure 2). For example, *TDRD1 *was significantly upregulated in *TMPRSS2-ERG *positive tumors compared to normal (*p *= 1.77*10^-9^, FC = 26) and fusion-negative samples (*p *= 1.51*10^-9^, FC = 23) whereas no remarkable difference between fusion-negative and normal prostate tissues was found (*p *= 0.025, FC = 1.1; Figure 3A). *CRISP3 *has been frequently suggested to be a biomarker for prostate cancer detection and prognosis [23-26] and showed the highest fold-change in the initial tumor-normal comparison (Additional file 3: Table S3). Inclusion of the *TMPRSS2-ERG *subgroup information, however, revealed that *CRISP3 *gene expression is associated with the *ERG *status, since it is significantly upregulated in *TMPRSS2-ERG *positive tumors compared to normal tissue (*p *= 2.2*10^-12^, FC = 37, or *p *< 0.001, FC = 29, respectively; Figure 3B). In contrast, less remarkable expression changes of *CRISP3 *were found between normal and fusion-negative tumor samples (*p *= 0.03, FC = 1.2; Figure 3B). To verify these results, we performed a technical validation in the same sample set by quantitative RT-PCR (Additional file 5: Figure S1) and we also included an independent validation study of recently published prostate cancer gene expression profiles [20]. *CRISP3 *and *TDRD1 *were also found to be considerably upregulated in gene fusion-positive samples compared to benign and fusion-negative tumor samples with qPCR-based analysis (Additional file 5: Figure S1) as well as in the validation cohort (Additional file 6: Figure S2).

The overlap between *TMPRSS2-ERG *deregulated genes and candidates for prostate cancer detection (Figure 2) might be due to the high expression fold-changes induced by ERG. *CACNA1D*, for instance, has been described as a downstream target of ERG, but was also found to be one of the top candidate for prostate cancer detection in the initial tumor--normal comparison. In line with previous reports, *MYO6 *was also found to be upregulated in *TMPRSS2-ERG *gene fusion positive tumors [27], although *MYO6 *has been reported as a marker for prostate cancer development [28]. Therefore, the results suggest that well-known biomarkers like *CRISP3 *and *MYO6 *might not be related to prostate cancer development but rather to transcriptional alterations induced by the overexpression of the transcription factor ERG in fusion positive prostate tumors. Other markers with unknown function (e.g. *TDRD1, F5) *might be associated with the molecular mechanism of *TMPRSS2-ERG *gene fusion, but should be avoided as biomarkers for prostate cancer, since they are not appropriate for the detection of *TMPRSS2-ERG *negative tumors.

### Activation of WNT and TGF-β/BMP signaling pathways in fusion-positive prostate cancer patients

To study the transcriptional changes induced by *TMPRSS2-ERG *gene fusion, we applied Ingenuity Pathway Analysis software to investigate overrepresented pathways among the genes with significant upregulation in fusion-positive tumors. The top canonical pathway was "Factors promoting cardiogenesis", a combination of TGF-β, BMP and WNT signaling (Additional file 7: Figure S3). Overrepresented genes from the top canonical pathway were validated in the publicly available data set of gene expression profiles from clinical prostate tumors [20]. Almost all of these pathway-specific genes were also found to be significantly upregulated in fusion-positive patients in the validation cohort (Table 1). Activated WNT signaling was recently described to be among the most highly enriched pathways in ERG-overexpressing tumors [13]. It is also associated with epithelial-to-mesenchymal transition in fusion-positive tumors [12]. In addition to WNT-signaling, we identified a significant upregulation of the TGF-β/BMP signaling pathways in *TMPRSS2-ERG *fusion-positive patients. The interaction between the pathways seems to be relevant in gene fusion-positive prostate cancer, since TGF-β has been extensively discussed as the main initiator of EMT [29] and is known to closely interact with WNT-signaling [30].

**Table 1:** Deregulated WNT, TGF-β and BMP signaling in fusion-positive tumors:

	Own Data		**Taylor et al. **[20]	
**GeneSymbol**	**FC**	***p *value**	**FC**	***p *value**

*ACVR1*	1.31	0.004	1.15	0.005

*ACVR2B*	1.33	< 0.001	1.11	0.007

*BMP7*	1.26	< 0.001	1.11	0.010

*BMPR2*	1.50	0.002	1.14	0.028

*FZD3*	1.72	< 0.001	1.28	0.011

*FZD5*	1.67	< 0.001	1.32	< 0.001

*FZD7*	1.29	0.005	1.12	0.442

*FZD8*	1.13	< 0.001	1.14	< 0.001

*LRP1*	1.89	< 0.001	1.35	< 0.001

*LRP6*	1.46	< 0.001	1.14	0.011

*MAP3K7*	1.42	< 0.001	1.17	0.005

*PRKCH*	1.25	0.002	1.09	0.074

*PRKD1*	1.86	< 0.001	1.47	< 0.001

*SMAD2*	1.45	0.005	1.18	0.006

*SMAD5*	1.50	< 0.001	1.28	< 0.001

*SMAD9*	1.29	0.002	1.20	< 0.001

*TCF7L2*	1.33	< 0.001	1.09	0.250

*TGFBR3*	1.44	< 0.001	1.11	0.231

Deregulated genes from the top canonical pathway ("Factors promoting cardiogenesis") according to the overrepresentation analysis using Ingenuity Pathway Analysis software

The exact molecular mechanisms leading to an enhancement of TGF-β signaling in fusion-positive tumors are so far unclear and should be analyzed in future studies. One explanation might be the cross-talk between androgen and TGF-β signalling [31]. ERG has recently been described to suppress androgen signalling [14]. Since androgen deprivation induces TGF-β signaling in prostate cells [32-34], it is tempting to speculate that ERG-induced suppression of androgen signal transduction leads to an increase of TGF-β pathway utilization which - in combination with WNT signalling - results in EMT and cell invasion in fusion-positive tumors.

## Conclusions

In conclusion, our data suggest that the *TMPRSS2-ERG *gene fusion marks a molecularly distinct tumor entity with substantial transcriptional modulation. In particular, our gene expression data indicate a deregulation of WNT and TGF-β/BMP signaling in fusion-positive prostate tumors.

The inclusion of benign samples in our gene expression analysis additionally demonstrated that well-known biomarkers for prostate cancer like *CRISP3 *are associated with the *TMPRSS2-ERG *fusion status. Thus, these genes might not be primarily related to the tumor status but rather to ERG-induced transcription remodeling. Therefore, it would be beneficial to consider the fusion status in the discovery and assessment of molecular biomarkers for prostate cancer. Recently, Karnes et al. were the first to test the performance of prostate cancer biomarkers in *TMPRSS2-ERG *positive and negative subgroups [35]. Their results underlined that the prior knowledge about the *TMPRSS2-ERG *fusion status will help to identify more accurate biomarkers and to develop novel targeted therapy strategies for prostate cancer in the future.

## Competing interests

The authors declare that they have no competing interests.

## Authors' contributions

JCB, RK, MM carried out the large-scale gene expression analysis using microarrays, MJ, MF, JCB analyzed the gene expression data; LAK, TA, JM performed validation experiments; MJ, MF, SG, performed statistical analysis and TB was responsible for the supervision of the analysis; TS, GS, RS, HSi collected clinical samples and data; HS, JCB, HM, UK, TS, RS, GS, were involved in the conceptual design of the study; HS was responsible for the supervision of the project, JCB and HS wrote the manuscript. All authors read and approved the final manuscript

## Pre-publication history

The pre-publication history for this paper can be accessed here:

http://www.biomedcentral.com/1471-2407/11/507/prepub

## Supplementary Material

Additional file 1 **Table S1**. Clinical information for the analyzed prostate cancer patient cohort. Click here for file

Additional file 2 **Table S2**. *TMPRSS2-ERG *gene fusion validation results: qualitative and quantitative RT-PCR reactions were carried out as described in materials and methods. The expression levels of *ERG *exons 2 and 3 were compared with the adjacent exons ("Exon walking plot") similar to the method described by Jhavar et al. [16]. *ERG *expression levels were normalized to the median expression in benign samples. Click here for file

Additional file 3 **Table S3**. Differentially expressed genes between 48 benign and 47 tumor samples with at least 2-fold expression changes as determined by modified t-statistic (FDR ≤ 0.05). Click here for file

Additional file 4 **Table S4**. Differentially expressed genes between 17 fusion-positive and 20 fusion-negative tumor samples with at least 2-fold expression changes as determined by modified t-statistic (FDR ≤ 0.05). Click here for file

Additional file 5 **Figure S1**. Association between prostate cancer biomarkers and *TMPRSS2-ERG *gene fusion as detected by q-RT PCR. Gene expression of *TDRD1 *(a) and *CRISP3 *(b) quantified by qRT-PCR in 48 normal and 20 fusion-negative and 17 fusion-positive tumors samples. Click here for file

Additional file 6 **Figure S2**. Association between prostate cancer biomarkers and *TMPRSS2-ERG *gene fusion in the validation cohort. Gene expression of *TDRD1 *(a) and *CRISP3 *(b) in 29 benign and 72 fusion-negative and 48 fusion-positive tumor samples. Click here for file

Additional file 7 **Figure S3**. Deregulated WNT/TGF-β/BMP signaling in fusion-positive tumors. Top canonical pathway ("Factors promoting cardiogenesis") of the overrepresentation analysis using the significantly upregulated genes in fusion-positive tumors. Deregulated genes of the specific pathway are indicated in grey. Click here for file
